# CXCR2 Blockade Mitigates Neural Cell Injury Following Preclinical Chorioamnionitis

**DOI:** 10.3389/fphys.2019.00324

**Published:** 2019-04-02

**Authors:** Tracylyn R. Yellowhair, Jessie C. Newville, Shahani Noor, Jessie R. Maxwell, Erin D. Milligan, Shenandoah Robinson, Lauren L. Jantzie

**Affiliations:** ^1^Department of Pediatrics, School of Medicine, The University of New Mexico, Albuquerque, NM, United States; ^2^Department of Neurosciences, School of Medicine, The University of New Mexico, Albuquerque, NM, United States; ^3^Division of Pediatric Neurosurgery, Department of Neurosurgery, The Johns Hopkins University School of Medicine, Baltimore, MD, United States

**Keywords:** preterm, chemokine, CXCL1, diffusion tensor imaging, neutrophil, white matter, alpha-II spectrin

## Abstract

Minimizing central nervous system (CNS) injury from preterm birth depends upon identification of the critical pathways that underlie essential neurodevelopmental and CNS pathophysiology. While chorioamnionitis (CHORIO), is a leading cause of preterm birth, the precise mechanism linking prenatal brain injury and long-term CNS injury is unknown. The chemokine (C-X-C motif) ligand 1 (CXCL1) and its cognate receptor, CXCR2, are implicated in a variety of uterine and neuropathologies, however, their role in CNS injury associated with preterm birth is poorly defined. To evaluate the putative efficacy of CXCR2 blockade in neural repair secondary to CHORIO, we tested the hypothesis that transient postnatal CXCR2 antagonism would reduce neutrophil activation and mitigate cerebral microstructural injury in rats. To this end, a laparotomy was performed on embryonic day 18 (E18) in Sprague Dawley rats, with uterine arteries transiently occluded for 60 min, and lipopolysaccharide (LPS, 4 μg/sac) injected into each amniotic sac. SB225002, a CXCR2 antagonist (3 mg/kg), was administered intraperitoneally from postnatal day 1 (P1)-P5. Brains were collected on P7 and P21 and analyzed with western blot, immunohistochemistry and *ex vivo* diffusion tensor imaging (DTI). Results demonstrate that transient CXCR2 blockade reduced cerebral neutrophil activation (myeloperoxidase expression/MPO) and mitigated connexin43 expression, indicative of reduced neuroinflammation at P7 (*p* < 0.05 for all). CXCR2 blockade also reduced alpha II-spectrin calpain-mediated cleavage, improved pNF/NF ratio, and minimized Iba1 and GFAP expression consistent with improved neuronal and axonal health and reduced gliosis at P21. Importantly, DTI revealed diffuse white matter injury and decreased microstructural integrity following CHORIO as indicated by lower fractional anisotropy (FA) and elevated radial diffusivity (RD) in major white matter tracts (*p* < 0.05). Early postnatal CXCR2 blockade also reduced microstructural abnormalities in white matter and hippocampus at P21 (*p* < 0.05). Together, these data indicate that transient postnatal blockade of CXCR2 ameliorates perinatal abnormalities in inflammatory signaling, and facilitates neural repair following CHORIO. Further characterization of neuroinflammatory signaling, specifically via CXCL1/CXCR2 through the placental-fetal-brain axis, may clarify stratification of brain injury following preterm birth, and improve use of targeted interventions in this highly vulnerable patient population.

## Introduction

Perinatal brain injury (PBI) is a major contributor to long-term disability in children across the globe (Blencowe et al., 2012, 2013; Kochanek et al., 2012). For a large proportion of infants with PBI, central nervous system (CNS) injury begins *in utero* secondary to inflammation (chorioamnionitis/CHORIO) and/or hypoxia-ischemia (HI) with placental insufficiency (Dammann and Leviton, 1997, 2014; Goldenberg et al., 2000; Lee J. et al., 2013; Lee S.M. et al., 2013; Chau et al., 2014; Fant et al., 2014). Specifically defined as infection/inflammation of the amniotic fluid, membranes, and placenta, concomitant with neutrophil infiltration into the choriodecidua along a chemotactic gradient of pro-inflammatory chemokines, (Yanowitz et al., 2002; Lee J. et al., 2013; Lee S.M. et al., 2013; Kallapur et al., 2014; Kim et al., 2015) CHORIO creates a toxic *in utero* microenvironment that limits oxygen exchange and propagates inflammation during critical periods of neurodevelopment (Redline, 2009, 2013; Galinsky et al., 2013; Anblagan et al., 2016). Typically, infants with PBI present with injury to major white and gray matter structures, leading to reduced connectivity of developing networks. Subsequently, diverse functional deficits ensue with impairment in multiple motor, cognitive and emotional realms, including educational under underachievement in childhood (Counsell et al., 2008; Boardman et al., 2010).

While CHORIO is implicated in preterm CNS injury, the molecular mechanisms meadiating inflammation in the placental-fetal-brain axis that causes PBI remains a gap in knowledge. Specifically, the overlap between placental and CNS physiology and the bi-directional cross talk between the developing immune system and neurodevelopment is relatively unknown. In pregnancy, the physiologic roles for chemokines are well described and dysregulated cytokine production due to infection has tremendous impact on the developing fetus (Hagberg and Mallard, 2005; Bastek et al., 2011; Hagberg et al., 2015). Notably, the chemokine (C-X-C motif) ligand 1 (CXCL1) and its cognate receptor (CXCR2) have been clinically implicated in the pathophysiology of CHORIO (Hsu et al., 1998; Lockwood et al., 2006; Bergeron et al., 2016). CXCL1 provides a chemotactic gradient for neutrophil infiltration to the maternal-fetal interface, and is extensively upregulated with intrauterine inflammation (Hsu et al., 1998; Lockwood et al., 2006). CXCL1 is also a major player in pregnancy failure from CHORIO (Saini et al., 2011; Mizugishi et al., 2015). Indeed, the severity of pathologic placental inflammation correlates positively with CXCL1 levels in newborns with CHORIO and funisitis, (Bry et al., 2015) and CXCL1 is upregulated in amniotic fluid, umbilical cord, and maternal plasma in both term and preterm babies with amniotic infection (Cohen et al., 1996). Pregnant women with intra-amniotic infection have significantly higher amniotic fluid concentrations of CXCL1, (Hsu et al., 1998) and high CXCL1 levels correlate with maternal and newborn peripheral white blood cell counts (Cohen et al., 1996).

In the brain, chemokine receptors play a crucial role in the onset, regulation, and propagation of inflammation. They are also essential in cellular communication, neuronal survival, and neural transmission (Reaux-Le Goazigo et al., 2013; Xu et al., 2017). CXCR2 is one of the most well characterized chemokine receptors, and is located at the cell surface and in the cytoplasm (Semple et al., 2010b; Veenstra and Ransohoff, 2012; Cao et al., 2014; Xu et al., 2017). CXCL1 is the dominant CXCR2 ligand expressed in the inflamed CNS, and its levels are directly proportional to its function (Kielian et al., 2001; Carlson et al., 2008; Kerstetter et al., 2009; Liu et al., 2010; Roy et al., 2012; Veenstra and Ransohoff, 2012). Signaling through CXCR2 is a non-redundant driving force for neutrophil recruitment from blood (Semple et al., 2010a). CXCR2 is also expressed on oligodendrocyte progenitors (OPCs) and microglia in the fetal brains as early as 19–22 weeks gestation (Filipovic et al., 2003), and CXCR2 activation by CXCL1 on OPCs regulates their proliferation and migration (Robinson et al., 1998; Robinson and Franic, 2001). Additionally, CXCR2 is constitutively expressed on other neural cells including neurons, astrocytes, (Filipovic et al., 2003) and monocytes (Valles et al., 2006; Lindner et al., 2008; Veenstra and Ransohoff, 2012). Multiple reports have suggested a role for aberrant CXCL1/CXCR2 signaling in adult CNS injury including stroke, traumatic brain injury (TBI), temporal lobe epilepsy, (Xu et al., 2017) neuropathic nociception, (Abbadie et al., 2009; Yang et al., 2016) central sensitization, (Zhang et al., 2013; Manjavachi et al., 2014) and mechanical hypersensitivity (Chen et al., 2018). Despite the wealth of scientific knowledge of CXCL1/CXCR2 pathophysiology in the mature CNS, their specific role in the pathophysiology in PBI is undefined.

Previously, we published that upregulation of CXCL1 commencing *in utero* negatively affects the fetal microenvironment and trajectory of CNS development (Jantzie et al., 2014a, 2018; Maxwell et al., 2015; Yellowhair et al., 2018). Specifically, CXCL1/CXCR2 signaling is increased following CHORIO in rat placenta, fetal and neonatal circulation and brain over an extended developmental time course, concomitant with increased numbers of placental and cerebral CXCR2-positive neutrophils, and other markers of neuroinflammation (Yellowhair et al., 2018). Thus, to evaluate the putative efficacy of CXCR2 blockade in neural repair following CHORIO, we tested the hypothesis that transient postnatal CXCR2 antagonism would reduce neutrophil activation, mitigate inflammation and neural injury, and preserve brain diffusion and microstructure following CHORIO in rats. The goal of this investigation was to connect aberrant CXCL1/CXCR2 signaling to PBI secondary to CHORIO, and putatively define novel targets for directed therapies for infants at high risk for PBI from CHORIO and related etiologies.

## Materials and Methods

### Animals

All procedures were performed consistent with National Research Council guidelines, and with the approval of the Institutional Animal Care and Use Committee (IACUC) at the University of New Mexico Health Sciences Center. ARRIVE guidelines were followed.

### In Utero Chorioamnionitis (CHORIO)

**FIGURE 1:**
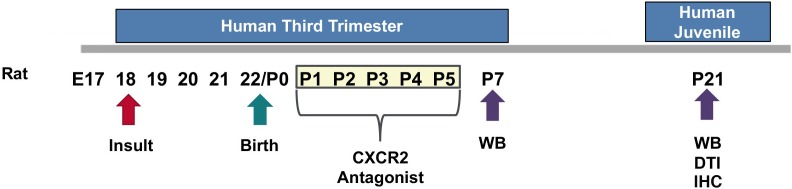
Experimental Design. On embryonic day 18 (E18), a prenatal chorioamnionitis insult was induced in pregnant Sprague Dawley rats. Rat pups were born on E22. SB225002, a CXCR2 antagonist, or vehicle, was administered during a critical neonatal window from postnatal day 1 (P1) through P5 (3 mg/kg i.p.). Pup brains were collected and analyzed on P7 or P21 for western blot analysis (WB), *ex vivo* diffusion tensor imaging (DTI), and immunohistochemistry (IHC).

We used an established model of CHORIO that yields deficits in the mature CNS that mimic those of preterm survivors (Jantzie et al., 2014a, 2018; Maxwell et al., 2015; Yellowhair et al., 2018). Specifically, pregnant Sprague Dawley rats underwent abdominal laparotomy on embryonic day 18 (E18), consistent with previous reports (Jantzie et al., 2013, 2014a,b, 2015a,b, 2018; Maxwell et al., 2015; Yellowhair et al., 2018). Uterine arteries were transiently occluded for 60 min, to induce placental insufficiency, followed by an intra-amniotic injection of lipopolysaccharide (LPS 0111:B4, 4 μg/sac; Sigma-Aldrich, St. Louis, MO, United States). Laparotomies were closed, and the rat pups were born at term on embryonic day 22 (E22). Sham dams underwent anesthesia for an equivalent duration of time without further intervention. Pups were then euthanized on postnatal day (P) 7 or P21 for biochemical or *ex vivo* magnetic resonance imaging (MRI) analyses. Previously, we reported the placental pathology, Fetal Inflammatory Response Syndrome (FIRS), neuroinflammatory responses, as well as MRI outcomes and the long-term cognitive and motor functional abnormalities in this model (Jantzie et al., 2014a, 2018; Maxwell et al., 2015; Yellowhair et al., 2018). For each experiment described, equal numbers of male and female pups were used in each assay, and data represents true n (individual pups) from at least four different dams per condition. A summary diagram of our experimental method is provided as Figure 1.

### Neonatal Administration of SB225002, a CXCR2 Antagonist

The selective, competitive CXCR2 antagonist SB225002 (Cayman Chemical, Ann Arbor, MI, United States) was used to block CXCR2 in rats following CHORIO. Previously, SB225002 has been reported to be safe in the developing CNS and has been widely used in adult models of CNS injury (Cao et al., 2014; Wang et al., 2016; Xu et al., 2017). Accordingly, CHORIO rat pups of both sexes were randomly assigned to treatment with SB225002 3 mg/kg (Cao et al., 2014; Wang et al., 2016; Xu et al., 2017) intraperitoneally (i.p.) from P1–P5, consistent with prior reports of translatable neonatal neurorepair dosing intervals with other compounds such as erythropoietin and melatonin (Figure 1; Jantzie et al., 2013, 2014b, 2015a,b, 2018) SB225002 was prepared by dissolving 25 mg of crystalline solid in 1 mL of DMSO. It was then aliquoted and stored at −20°C. On the day of experimentation, frozen SB225002 stock was further diluted to a working concentration of 0.5 mg/mL in 30% DMSO and 70% normal saline, after which a 3 mg/kg dose was prepared for each pup using normal saline. The total injection volume for each injection was 100 μl. Sham rat pups received a vehicle injection of 30% DMSO and 70% normal saline solution only.

### Western Blot

Microdissected cortical samples from sham, CHORIO, or CHORIO rats treated with CXCR2 antagonist at P7 or P21 were homogenized and sonicated, and centrifuged at 4200 × *g* for 10 min consistent with prior reports (Jantzie et al., 2015a,b,c, 2016). Protein concentration in the whole cell fraction was determined with a Bradford assay (Bio-Rad, Hercules, CA, United States). Thirty micrograms of protein were loaded on 4–20% Tris–HCl gels or 4–12% bis-tris HCl gels (Bio-Rad), separated by electrophoresis, and transferred to polyvinylidene fluoride (PVDF) membranes. Membranes were blocked with 5% non-fat dry milk in TBST and incubated with primary antibody overnight at 4 degrees. A species appropriate horseradish-peroxidase-conjugated secondary antibody (Thermo, Grand Island, NY, United States) was applied, and after washing, detected with chemiluminescence (Thermo) using a LAS 4000 imager (GE, Healthcare, PA). Primary antibodies against the following targets were used consistent with prior publications: alpha-II spectrin (Santa Cruz, Dallas, TX, 1:100), myeloperoxidase (MPO) (AbCam, Cambridge, MA, 1:500, connexin43 (CX43, Cell Signaling, Danvers, MA, 1:500), phosphoneurofilament (pNF, Millipore, Temecula, CA, 1:500) or neurofilament (NF, SMI-312, Covance, Princeton, NJ, 1:1000) (Jantzie et al., 2016; Yellowhair et al., 2018). Blots were imaged using an ImageQuant LAS 4000 (GE) and bands of interest were quantified using ImageQuant Software (GE) normalized to the loading control, actin (Sigma, St. Louis, MO, 1:5000). At least two blots were used to assay each protein. Data was then normalized to the sham group consistent with previous reports (Veenstra and Ransohoff, 2012; Jantzie et al., 2014b, 2015a, 2016).

### Immunohistochemistry

On P21 rats were deeply anesthetized with sodium pentobarbital and perfused with 4% paraformaldehyde. Brains were then collected, and post-fixed in paraformaldehyde. After immersion in 30% sucrose solution, 20 μm, frozen, slide mounted, coronal sections were obtained and collected using a cryostat (Leica, Buffalo Grove, IL, United States). Slides were then washed and incubated with 0.3% hydrogen peroxide, followed by blocking solution containing 10% normal goat serum in phosphate buffered solution (PBS). Primary antibodies against glial fibrillary acidic protein (GFAP, Dako 1:500, Carpinteria, CA, United States) or ionized calcium binding adaptor 1 (Iba1, Wako, 1:500, United States), in blocking solution containing 0.1% Trition-X100 were incubated on sections overnight at 4°C. The next day, sections were rinsed, and incubated with species-appropraite biotinylated secondary antibodies for 1 h. This was followed by incubation in VECTASTAIN (Vector Labs, Burlingame, CA, United States) and 3,3′-diaminobenzidine (DAB). Sections were then processed for dehydration, cleared in xylenes and cover-slipped in Permount (Millipore Sigma, St. Louis, MO, United States). Appropriate negative controls without primary antibodies were run in parallel. Using bright-field illumination, representative images were photographed on an upright Leica microscope.

### Stereological Estimates

All P21 sections were coded prior to analyses by a blinded observer and stereology performed consistent with previously published methodology (Jantzie et al., 2014a, 2015a; Robinson et al., 2017a). Estimates of the load of each antigen were obtained from 20 μm coronal sections using a thin section modification of the optical fractionator method (Gundersen et al., 1988; Mouton et al., 2002). Specifically, object area fraction and volume probes (Cavalieri’s method) were used to calculate load and to quantify the amount of Iba1-positive microglia and GFAP-positive astrocytes in the fimbria. At the completion of the stereological analyses, the samples were decoded, and mean and SEM of load were calculated.

### Diffusion Tensor Imaging (DTI)

*Ex vivo* MRI using diffusion sequences was performed on a Bruker BioSpec 7T 70/30 Ultra Shield Refrigerated (USR) nuclear MRI system, consistent with prior published methods (Robinson et al., 2016, 2017b; Yellowhair et al., 2018). Briefly, P21 rats were deeply anesthetized with sodium pentobarbital and perfused with 4% paraformaldehyde. Brains were removed from the skull and post-fixed in 4% paraformaldehyde for 1 week and embedded in 2% agarose containing 3 mM sodium azide for immediate *ex vivo* MR imaging. Echo-planar diffusion tensor imaging (EP-DTI) of twenty contiguous coronal 1 mm slices were obtained with a FOV (field-of-view) of 3.00 cm and an MTX of 256. Brain regions of interest (ROI) in major white matter tracts (corpus callosum and external capsule) and gray matter (hippocampus and thalamus), were traced by an observer blinded to experimental conditions and analyzed using Bruker’s ParaVision 5.1 imaging software. Fractional anisotropy (FA), axial (λ1) and radial [(λ2+λ3)/2] diffusivity eigenvectors were measured and calculated. For bilateral neuroanatomical ROIs, metrics were acquired on each side and averaged per ROI. Directionally encoded diffusion color maps and color-coded FA maps were created.

### Statistical Analyses

Data are represented as mean ± the standard error of the mean (SEM). Parametric statistical differences between three groups (sham, CHORIO and CHORIO+CXCR2 antagonist) were established using a two-way ANOVA with Bonferroni *post hoc* correction to discern the effects of injury and treatment. *p* < 0.05 was considered statistically significant.

## Results

### Transient Blockade of CXCR2 Attenuates Neutrophil Activation and Reduces Connexin43 Expression

**FIGURE 2:**
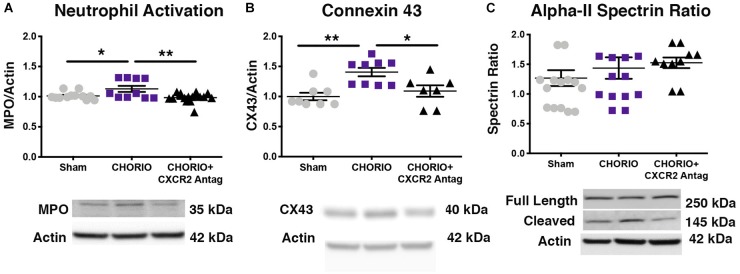
CXCR2 Antagonism Reduces Neutrophil Activation and Connexin43 Expression on Postnatal Day 7 (P7). Following prenatal chorioamnionitis, transient postnatal administration of the CXCR2 antagonist, SB225002, reduced neutrophil reactivity (myeloperoxidase, MPO, **A**) and mitigated connexin43 expression (CX43, **B**) in the cortex on P7, consistent with reduced cellular neuroinflammation. At P7, neither CHORIO or CXCR2 antagonism changed cleaved alpha-II spectrin ratio, a marker of neuronal health **(C)**. (*n* = 7–15, two-way ANOVA with Bonferroni’s *post hoc* correction, ^∗^*p* < 0.05, ^∗∗^*p* < 0.01).

To first establish putative beneficial effect of CXCR2 blockade on neutrophil activation, a common cellular mediator of inflammation throughout the placental-fetal-brain axis, we examined cerebral myeloperoxidase (MPO) protein expression on P7, 48 h following the last dose of SB225002. As expected, vehicle-treated CHORIO pups had increased MPO expression compared shams (*n* = 10–15, *p* < 0.05, Figure 2A). Notably, treatment with SB225002, the CXCR2 antagonist, mitigated neutrophil activation in CHORIO pups and restored MPO protein expression levels to that observed in sham pups (*p* < 0.01, Figure 2A).

As CXCR2 has been documented to be important in intracellular signaling and communication under normal and inflamed conditions, we also examined the effect of CXCR2 blockade on connexin43 expression, a hemichannel protein that is present in placenta and brain, including on astrocytes and other immune cells (Dunk et al., 2012; Theodoric et al., 2012; Chen et al., 2014; Yin et al., 2018). Indeed, connexin43 is a gap junction protein intimately connected to CXCR2 activation, astrocyte activation, and excitotoxicity (Theodoric et al., 2012; Chen et al., 2014). Consistent with increased neuroinflammatory signal transduction in our model of CHORIO, (Jantzie et al., 2014a; Yellowhair et al., 2018) connexin43 protein expression was significantly elevated in the brains of vehicle-treated CHORIO pups at P7 (*n* = 7–9, *p* < 0.01, Figure 2B). Similar to the reduction in MPO observed with CXCR2 antagonism, treatment with SB225002 also significantly reduced connexin43 expression at P7 (*p* < 0.05, Figure 2B). We also examined alpha-II spectrin at P7, a neuron specific cytoskeletal protein and target of calpain (Jantzie et al., 2014b, 2016; Schober et al., 2014). Consistent with our previous reports, we found no effect of CHORIO or SB225002 administration at P7 on the ratio of full length alpha-II spectrin to cleaved alpha-II spectrin (*n* = 10–16 *p* = ns, Figure 2C).

### CXCR2 Antagonism Attenuates CHORIO-Induced Gliosis and Neural Injury

**FIGURE 3:**
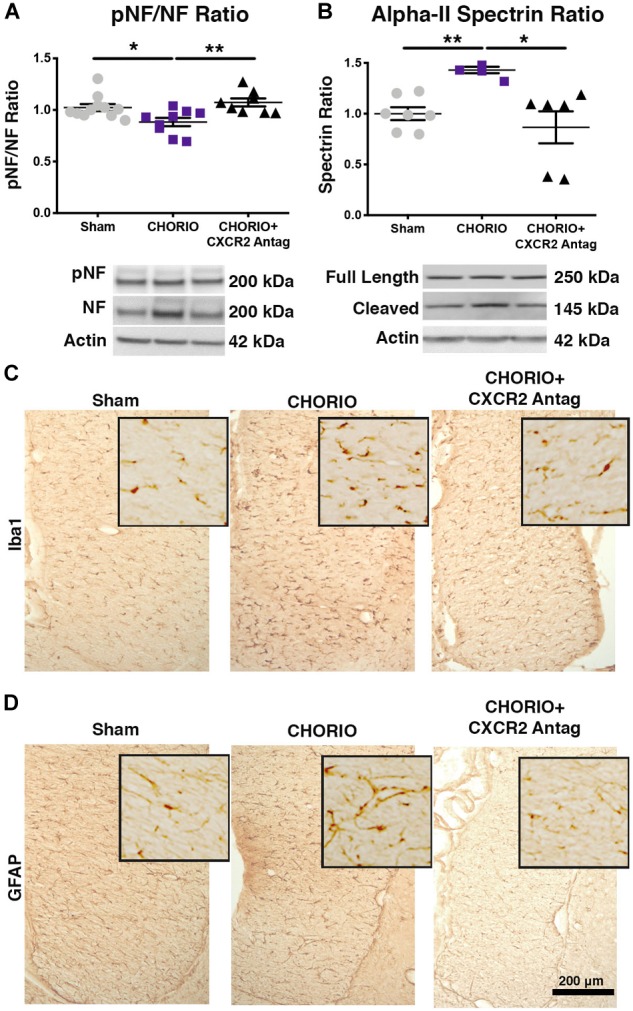
CXCR2 Antagonism Improves Axonal and Neuronal Health Through Postnatal Day 21 (P21). Following prenatal chorioamnionitis (CHORIO), transient postnatal administration of the CXCR2 antagonist, SB225002, mitigated injury-induced alterations in cortical phosphoneurofilament to neurofilament ratio (pNF/NF, **A**), consistent with improved axonal health. Similarly, transient CXCR2 blockade also reduced pathological augmentation of alpha-II spectrin cleavage in the cortex **(B)**, consistent with reduced calpain activity and improved neuronal health. Immunoreactivity for microglia (Iba1, **C**) and astrocytes (GFAP, **D**) is augmented at P21 in CHORIO fimbria compared to Sham, an effect that is ameliorated by CXCR2 antagonism. Scale bar = 200 μm. (*n* = 6–11/group for immunoblot, *n* = 3 for immunohistochemistry, two-way ANOVA with Bonferroni’s *post hoc* correction, ^∗^*p* < 0.05, ^∗∗^*p* < 0.01).

Given the acute effect of CXCR2 blockade at P7 on neutrophil activation and reduced connexin43 expression, we examined global markers of axons, astrocytes, microglia and assessed neuronal health at P21 to establish longer term effects of CXCR2 blockade (Figure 3). P21 in rats is equivalent to a young, human juvenile and represents a timepoint 4 weeks following *in utero* insult and just over 2 weeks following the last dose of SB225002 (Semple et al., 2013; Jantzie and Robinson, 2015). Notably, transient neonatal antagonism of CXCR2 with SB225002 significantly improved the ratio of pNF to NF compared to CHORIO pups treated with vehicle, consistent with improved axonal health in treated pups and similar levels of these key axonal proteins as shams (*n* = 9–11, *p* < 0.01, Figure 3A). Given the beneficial effect of CXCR2 antagonism on axons, we then examined the alpha-II spectrin ratio in treated and untreated pups at P21. Importantly, the spectrin ratio in vehicle-treated CHORIO pups was elevated compared to shams (*n* = 6–7, *p* < 0.01, Figure 3B), consistent with elevated levels of calpain protease activity and neuronal cytoskeletal breakdown. Notably, treatment with the CXCR2 antagonist SB225002, mitigated the increase in spectrin cleavage ratio and normalized alpha-II spectrin cleavage to sham levels (*n* = 6–11, *p* < 0.05, Figure 3B). We also examined microglia and astrocytes at this time point. Immunoreactivity for microglia (Iba1, Figure 3C) and astrocytes (GFAP) (Figure 3D) was augmented at P21 in the fimbria of CHORIO animals compared to Sham, an effect that was ameliorated by CXCR2 antagonism. Using stereological principles, we quantified Iba1 and GFAP load (Table 1). These analyses confirmed significant increases in Iba1 and GFAP load induced by CHORIO that was significantly mitaged by CXCR2 antagonism. Together, these data indicated that transient CXCR2 antagonism attenuates gliosis, and reduces axonal and neuronal injury following CHORIO.

**Table 1:** Stereological Estimates of Iba1 and GFAP Load in the Fimbria.

	Sham	CHORIO	CHORIO+CXCR2 ANTAG
**Iba1 (μm**^3^**)**	4.9+1.2 × 10^6^	14.5+1.1 × 10^6∗∗^	7.2+0.3 × 10^6∗^
**GFAP (μm**^3^**)**	4.1+0.7 × 10^6^	15.1+1.7 × 10^6∗^	7.7+1.5 × 10^6∗^

*Asterix(s) in CHORIO column indicates statistical difference from Sham group. Asterix in CHORIO+CXCR2 ANTAG column indicates statistical difference from CHORIO group. ^∗^p < 0.05, ^∗∗^p < 0.01.*

### CXCR2 Blockade During a Critical Postnatal Window Mitigates the Effects of CHORIO and Protects the Developing Brain

**FIGURE 4:**
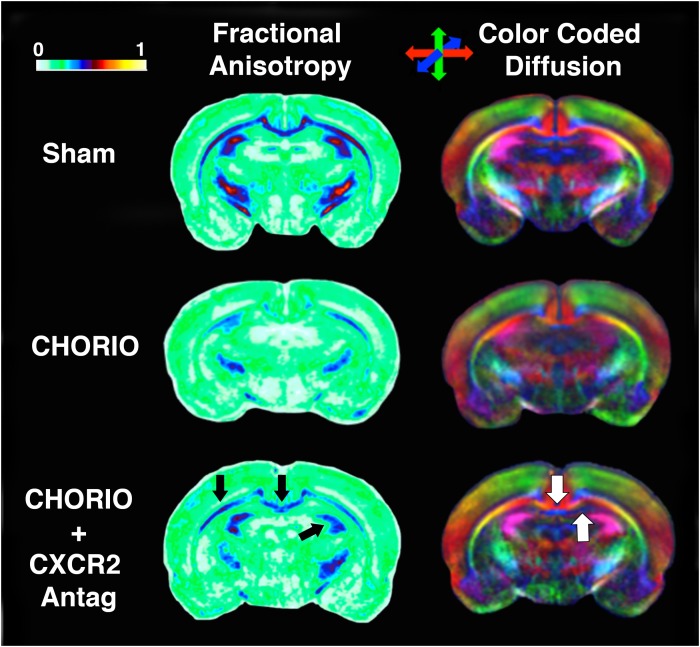
CXCR2 Antagonism Mitigates Abnormalities in Cerebral Diffusion. Following prenatal chorioamnionitis (CHORIO), transient postnatal administration of the CXCR2 antagonist, SB225002, attenuated losses of fractional anisotropy (FA) in major white matter tracts (left, black arrows) on postnatal day 21 (P21). Similarly, directionally encoded color maps (right) reveal loss of diffusion in white and gray matter, including the corpus callosum and anterior hippocampus (white arrows). Color coded FA legend (left) indicates the degree of anisotropy from 0 to 1, with 0 being unrestricted and 1 fully restricted. Directionally color encoded arrows (right) indicate horizontal diffusion (red), anterior to posterior diffusion (blue), and superior-inferior diffusion (green).

**FIGURE 5:**
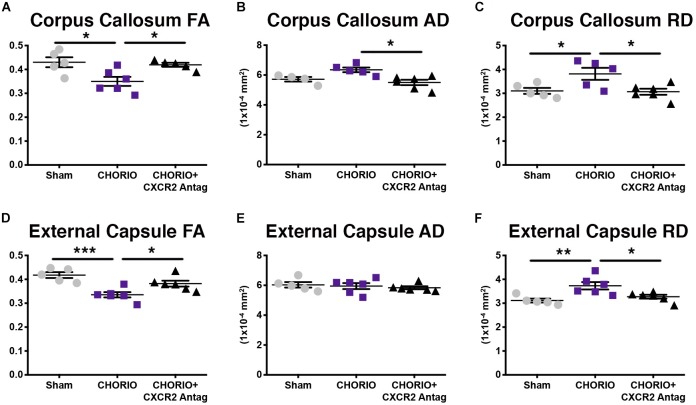
CXCR2 Antagonism Attenuates White Matter Microstructural Injury. Following prenatal chorioamnionitis (CHORIO), transient postnatal administration of the CXCR2 antagonist, SB225002, attenuated injury induced changes in fractional anisotropy **(A)** in the corpus callosum at postnatal day 21 (P21), together with mitigating changes in axial (AD, **B**) and radial (RD) diffusion **(C)**. Similarly, CHORIO animals had reduced FA **(D)** and increased RD **(F)** in the external capsule. Treatment with a CXCR2 antagonist significantly improved both FA and RD. No changes were observed in AD **(E)**. (*n* = 5–6, two-way ANOVA with Bonferroni’s *post hoc* correction, ^∗^*p* < 0.05, ^∗∗^*p* < 0.01, ^∗∗∗^*p* < 0.001).

**FIGURE 6:**
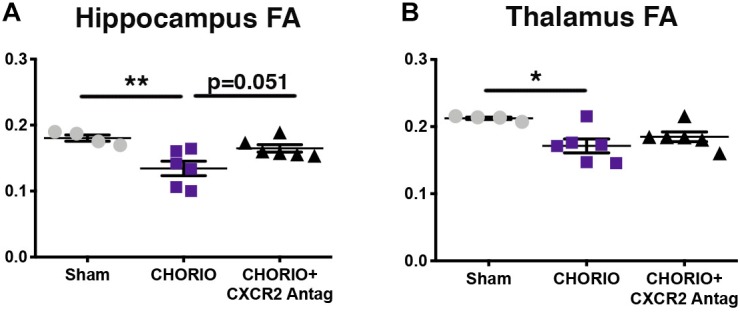
CXCR2 Antagonism Improves Gray Matter Microstructural Integrity. Following prenatal chorioamnionitis (CHORIO), transient postnatal administration of the CXCR2 antagonist, SB225002, attenuated injury induced changes in fractional anisotropy (FA) in the hippocampus **(A)** at postnatal day 21 (P21). While CHORIO reduced FA in the thalamus, no difference was observed with CXCR2 antagonism in the thalamus **(B)**. (*n* = 5–6, two-way ANOVA with Bonferroni’s *post hoc* correction, ^∗^*p* < 0.05, ^∗∗^*p* < 0.01).

Given the biochemical evidence of reduced neuroinflammation and neural health at P7, and at P21, we next performed DTI analyses to examine the effects of CXCR2 antagonism on white and gray matter microstructure using a translational imaging outcome measure. We began by creating color coded fractional anisotropy (FA) maps and directionally-encoded color diffusion maps (Figure 4). Both color FA and the directionally encoded color diffusion maps depict loss of structural integrity in major white matter tracts, including the corpus callosum, fimbria, external and internal capsule, and demonstrate subsequent improvement with CXCR2 antagonism (Figure 4). Indeed, quantification of diffusion metrics and scalars in corpus callosum (Figure 5A–C) and external capsule FA (Figure 5D–F), confirms significant loss of microstructural integrity in CHORIO pups that is recovered with CXCR2 antagonism. These changes in FA are also associated with injury-induced elevations in radial diffusivity (RD). Notably, CXCR2 antagonism ameliorated CHORIO-induced elevations in RD in both the corpus callosum and external capsule (Figure 5C,F), and restored axial diffusivity (AD) in the corpus callosum (Figure 5B), consistent with improved white matter and axonal health, and improved structural coherence and directional diffusion. We also examined gray matter microstructure using DTI. Similar to the white matter regions, we found injury induced decreases in hippocampal and thalamic FA in CHORIO pups compared to shams (*n* = 5–6/group, *p* < 0.01, Figure 6). Also consistent with white matter regions, administration of SB225002, restored FA in the hippocampus, indicative of improved gray matter microstructure. No changes in RD and AD were noted in the gray matter (data not shown).

## Discussion

Chemokines, a family of small molecular weight chemotactic cytokines, (Ben-Baruch et al., 1995; Luster, 1998; Kielian et al., 2001) are classically defined by their ability to induce directional migration and activation of leukocytes to areas of inflammation in the body (Kielian et al., 2001; Semple et al., 2010b; Veenstra and Ransohoff, 2012). Chemokine signaling is also integral to development of multiple placental and neural cell lineages. CXCL1 is an ERL (glutamic acid-arginine-leucine) CXC chemokine defined by potent CXCR2 receptor-dependent neutrophil chemoattractant activity, whereas CXC chemokines lacking the ERL motif are inactive toward neutrophils ( Murphy, 1997; Biondo et al., 2014). CXCL1 is the dominant CXCR2 ligand expressed in the inflamed CNS (Roy et al., 2012). CXCL1 transcripts are 4-fold more abundant in the brain than those of CXCL2 and its levels are directly proportional to its function (Roy et al., 2012). Upregulation of CXCL1/CXCR2 signaling is central to neuroinflammation following exposure to bacterial endotoxin, (Kielian et al., 2001; Carlson et al., 2008; Kerstetter et al., 2009; Liu et al., 2010; Roy et al., 2012) and CXCL1 is rapidly upregulated in traumatic brain injury (TBI), Alzheimer’s disease, multiple sclerosis, chronic pain and stroke, and is followed by neural cell specific increases in CXCR2 expression (Valles et al., 2006; Lindner et al., 2008; Kerstetter et al., 2009; Liu et al., 2010, 2015; Cao et al., 2014; Connell et al., 2015; Ryu et al., 2015). Indeed, CXCR2 expression is essential for cerebral endothelial activation and leukocyte recruitment, (Wu et al., 2015) with CXCR2 antagonism or CXCL1 deficiency mitigating neutrophil infiltration and recruitment into brain parenchyma (Wu et al., 2015). In alignment with these data and with clinical literature confirming that PBI in preterm infants often originates *in utero*, the present investigation supports the hypothesis that CXCL1/CXCR2 signaling negatively impacts brain development. Importantly, here we show CXCR2 blockade reversed injury-induced CNS elevations in neuron-specific alpha-II spectrin cleavage, an established biomarker of PBI (Jantzie et al., 2014b, 2016). CXCR2 blockade also mitigated connexin43 expression, a hemichannel and gap junction protein connected to CXCR2 activation, astrocyte activation, and excitotoxicity (Theodoric et al., 2012; Chen et al., 2014; Yin et al., 2018). Interestingly, activated astrocytes can release CXCL1 and facilitate CXCR2 signal transduction via connexin43 to enhance and feed forward neuroinflammation (Cao et al., 2014; Chen et al., 2014, 2018). Similarly, here CXCR2 blockade attenuated white matter loss and axonal injury and mitigated CHORIO-induced increases in Iba1 and GFAP expression, and provided sustained protection to white and gray matter microstructure 4 weeks following *in utero* exposure to CHORIO. These data validate CXCR2 blockade and a putative functional relationship between CXCL1/CXCR2 and neural injury *in vivo*, and support the hypothesis that CXCL1/CXCR2 signaling is a prominent mediator of inflammation through the placental-fetal-brain axis. Moreover, we provide first evidence of the primacy of excess CXCR2 activation in white and gray matter neural injury that hallmarks PBI.

Prior preclinical reports confirm that CXCR2 blockade may be beneficial in the mature CNS. CXCR2 is dysregulated on OPCs and monocytes/microglia during demyelination (Lindner et al., 2008). On neurons, CXCL1 induction sustains late-phase neuropathic pain by activating CXCR2, and modulates synaptic transmission (Chen et al., 2014). Blockade of CXCR2 with the same inhibitor used here, SB225002, reverses allodynia and suppresses injury-induced increases in neuronal firing frequency, confirming a role for CXCL1/CXCR2 in synaptic plasticity (Chen et al., 2014). In TBI and stroke, cerebral CXCL1/CXCR2 levels determine the magnitude of neutrophil infiltration, subsequent neuronal loss, and infarct volume (Semple et al., 2010a; Hennessy et al., 2015). Here, we show that CXCR2 blockade during a critical postnatal window reverses some of the key biochemical and imaging hallmarks of CHORIO through P21 and protects the developing brain from excess CXCR2 signaling. Together, with high-resolution DTI confirming that CXCR2 blockade also reduced microstructural white and gray matter injury and resolves pathological changes in diffusion, these data emphasize the role of CXCL1/CXCR2 signaling in PBI defined by *in utero* inflammation and for the first time report the putative efficacy of transient CXCR2 blockade in the developing brain. Interestingly, numerous studies in adult animals emphasize that blocking chemokine receptors such as CXCR2, rather than individual ligands such as CXCL1, is most effective for neural repair (Semple et al., 2010a,b; Veenstra and Ransohoff, 2012). While commonly used, CXCR2 knockout (KO) mice have drawbacks including lymph node enlargement and myelination defects, (Tsai et al., 2002; Cardona et al., 2008; Semple et al., 2010a,b) emphasizing the fundamental role for CXCR2 in normal physiology and neurodevelopment. We hypothesized that rather than complete CXCR2 silence throughout development, transient loss of excessive CXCR2 signaling following CHORIO would mitigate brain injury. Additional dose-response and duration-response studies beyond the scope here are needed to clarify the optimal dosing regimen for SB225002. Indeed, a gradient effect may be promising for therapeutics targeting CXCL1/CXCR2 because it suggests partial receptor inhibition may be sufficient to attenuate neutrophil activation and improve neural health without complete cessation of essential biological processes that may cause additional detrimental effects in the developing injured brain (Abdulkadir et al., 2010; Semple et al., 2010a).

Infants with CHORIO have elevated neutrophil and monocyte counts compared to infants without intra-amniotic infection, (Weitkamp et al., 2016) emphasizing compartment-specific modulation of cytokines and immune cell diversity in CHORIO. Previously, we have shown that elevated placental CXCL1 is associated with acute neutrophilia and immune cell activation in the placenta, and with neutrophilia, immune cell activation and microgliosis in the brain (Jantzie et al., 2014a; Maxwell et al., 2015; Yellowhair et al., 2018). Using flow cytometry, we also demonstrated increased CXCR2^+^ neutrophils in the placenta and brain following CHORIO (Yellowhair et al., 2018). Here, we show that CXCR2 blockade attenuates neutrophil activation in the brain at P7. Together with prior publications confirming a sustained neuroinflammatory response following CHORIO, hallmarked by upregulated CXCL1, and increased microglia and macrophages, (Jantzie et al., 2014a; Maxwell et al., 2015; Yellowhair et al., 2018) these data indicate that CXCR2 antagonism reduces GFAP and Iba1 immunoreactivity consistent with changes to microglia and astrocytes. While future investigations will examine microglial activation state and regional and temporal changes in glial morphology and number, these data are consistent with earlier reports demonstrating that cerebral CXCL1 and CXCR2 levels determine the magnitude of neutrophil infiltration, subsequent neuronal loss, gliosis and infarct volume in stroke and TBI (Semple et al., 2010a; Hennessy et al., 2015). Typically, neutrophil influx is a secondary response after injury, and further exacerbates acute endogenous brain inflammation mediated by microglia (Gelderblom et al., 2009; Jellema et al., 2013). Indeed, limiting neutrophil and macrophage infiltration improves neuropathological and functional outcomes in models of stroke and TBI (Semple et al., 2010a; Morganti et al., 2015). In preterm sheep with hypoxia-ischemia, neutrophils invade vulnerable brain regions, including hippocampus, periventricular and subcortical white matter, (Jellema et al., 2013) with mobilization and recruitment accompanied by prominent microgliosis (Raivich et al., 1999; Valles et al., 2006). Indeed, CXCR2 expression on neural cells is essential for cerebral endothelial activation and leukocyte recruitment, (Wu et al., 2015) with CXCR2 antagonism or CXCL1 deficiency mitigating neutrophil infiltration and recruitment into brain parenchyma, as well as microgliosis (Wu et al., 2015).

Despite including both sexes in all outcome measures of this study, we were underpowered to detect sex differences. Future investigations beyond the scope of the present study should include assessments at later time points with translational measures of behavior and function consistent with previous reports (Jantzie et al., 2018; Robinson et al., 2018). Another limitation is the dosing regimen of SB225002, and the duration and timing of dose-response will be the focus of future studies, as well as a complete neuropathological examination and with spatiotemporal regional assessment of oligodendrocyte, neuron, astrocyte and microglial number. Glial activation state and morphology should also be assessed rigorously. In conclusion, this is the first report that transient postnatal blockade of CXCR2 modulates PBI pathophysiology and attenuates neural injury following CHORIO. Moreover, we provide the first evidence of the primacy of excess CXCR2 activation in white and gray matter neural injury that hallmarks PBI. While blocking microglial activation, neutrophil activation and inflammatory signaling is beneficial after CHORIO, the homeostatic control of chemokine signaling during development, injury, and repair cannot be overemphasized. The blockade of receptors essential for normal physiological properties, such as CXCR2, warrants further investigation.

## Data Availability

All datasets generated for this study are included in the manuscript and/or the supplementary files.

## Author Contributions

LJ conceptualized the hypothesis, and supervised the experiments. TY, JN, SN, JM, EM, SR, and LJ designed and performed the experiments. SR and LJ interpreted the data. LJ and TY wrote the manuscript. All authors contributed to manuscript revision and approved the final version.

## Conflict of Interest Statement

The authors declare that the research was conducted in the absence of any commercial or financial relationships that could be construed as a potential conflict of interest.
